# Healthy *versus* Unhealthy Suppliers in Food Desert Neighborhoods: A Network Analysis of Corner Stores’ Food Supplier Networks

**DOI:** 10.3390/ijerph121214965

**Published:** 2015-11-30

**Authors:** Yeeli Mui, Bruce Y. Lee, Atif Adam, Anna Y. Kharmats, Nadine Budd, Claudia Nau, Joel Gittelsohn

**Affiliations:** 1 Global Obesity Prevention Center (GOPC) at Johns Hopkins University, 615 N. Wolfe Street, Baltimore, MD 21205, USA; brucelee@jhu.edu (B.Y.L.); aadam1@jhu.edu (A.A.); akharmats@jhu.edu (A.Y.K.); cnau1@jhu.edu (C.N.); jgittel1@jhu.edu (J.G.); 2 Center for Human Nutrition, Department of International Health, Johns Hopkins Bloomberg School of Public Health, 615 N. Wolfe Street, Baltimore, MD 21205, USA; nbudd1@jhu.edu

**Keywords:** food supply, obesity, healthy food availability, network analysis, corner store, food deserts, urban health, food environment

## Abstract

*Background*: Products in corner stores may be affected by the network of suppliers from which storeowners procure food and beverages. To date, this supplier network has not been well characterized. *Methods*: Using network analysis, we examined the connections between corner stores (*n* = 24) in food deserts of Baltimore City (MD, USA) and their food/beverage suppliers (*n* = 42), to determine how different store and supplier characteristics correlated. *Results*: Food and beverage suppliers fell into two categories: Those providing primarily healthy foods/beverages (*n* = 15) in the healthy supplier network (HSN) and those providing primarily unhealthy food/beverages (*n* = 41) in the unhealthy supplier network (UHSN). Corner store connections to suppliers in the UHSN were nearly two times greater (*t* = 5.23, *p* < 0.001), and key suppliers in the UHSN core were more diverse, compared to the HSN. The UHSN was significantly more cohesive and densely connected, with corner stores sharing a greater number of the same unhealthy suppliers, compared to HSN, which was less cohesive and sparsely connected (*t* = 5.82; *p* < 0.001). Compared to African Americans, Asian and Hispanic corner storeowners had on average −1.53 (*p* < 0.001) fewer connections to suppliers in the HSN (*p* < 0.001). *Conclusions*: Our findings indicate clear differences between corner stores’ HSN and UHSN. Addressing ethnic/cultural differences of storeowners may also be important to consider.

## 1. Introduction

Over the last decade, a line of studies has highlighted significant disparities in overweight and obesity by race, socioeconomic status, and the neighborhood food environment [1,2,3,4,5,6]. In the United States, more than 1 out of 3 adults and approximately 1 out of 5 adolescents are obese, with a continued rising trend in most age groups and the highest prevalence among minority populations [7,8]. Overweight and obesity are also strongly associated with comorbidities such as cardiovascular disease, type 2 diabetes, and some cancers [9,10], as well as with social stigma and discrimination which can further impair quality of life of an individual struggling with weight [11].

Research has reported that neighborhoods with low-income and predominantly African American residents have fewer supermarkets but more liquor stores, convenience stores, and corner stores compared to higher income and Caucasian neighborhoods [12,13,14,15]. Consequently, shoppers in such neighborhoods tend to purchase significantly more unhealthy products, such as sugar-sweetened beverages and high-fat snacks [4], compared to shoppers in other neighborhoods where there are fewer barriers to practicing healthy eating.

In many low-income urban settings, corner stores have been an important venue for providing healthier food options among some intervention trials, given its ubiquitous presence and function as a major food source for children and families in these areas. However, a key barrier to sustaining such food environment changes that has received less attention is storeowners’ limited access to healthy food suppliers. Efforts have instead focused on customer-centric point-of-purchase promotions, educational flyers, cooking demonstrations, and vouchers or other incentives to raise awareness and promote healthy food purchases [16,17,18,19]. Other researchers have designed workshops with storeowners, community organizations, and policymakers to collaboratively design and refine intervention strategies [20]. At the store level, trials have implemented health education and business training modules, including how to handle fresh produce and make structural improvements (e.g., refrigeration systems, display stands) [21,22]. In addition, interventions have included pricing strategies to reduce the cost of purchasing healthier food options at the consumer and store levels by providing vouchers or other incentives such as store loans [16,17,18]. While some progress has been reported in improving the local food environment as well as the purchase and consumption of healthy foods, sustainability of changes and long-term impact on obesity, have yet to be achieved [23]. Thus, researchers have increasingly shifted towards multi-level and systems approaches to more comprehensively understand and address the issue [24,25].

To our knowledge, no study has characterized storeowners’ supplier networks (*i.e.*, connections between corner stores and their range of suppliers) nor evaluated store characteristics (*i.e.*, storeowners’ race/ethnicity) that may be associated with a corner store’s supplier networks. Interviews with retailers about their stocking practices and beliefs related to the supply of foods revealed that supplier networks varied by product. Storeowners reported on convenient delivery by manufacturers for unhealthy products such as chips and soda, and far fewer connections with suppliers of healthy options such as fruits and vegetables, thus requiring storeowners to self-supply and stock [26]. In food environment research, greater understanding of these supplier networks is important because the connections and the type of suppliers accessed by storeowners may constrain the food and beverage offerings in stores. Corner stores that receive inventory from a select sub-set of suppliers may be more limited in what they can potentially stock. While food supply and distribution networks are well defined for larger food establishments, research on the supplier networks of smaller food stores is lacking. Characterizing and quantifying corner stores’ supplier networks can shed light on how to best develop public health programs and policies by addressing acquisition barriers experienced by storeowners.

## 2. Experimental Section

### 2.1. Study Setting

The corner stores included in this sample were originally part of a community trial, B’More Healthy Retail Rewards (BHRR) that was implemented in low-income areas of East and West Baltimore City, Maryland (MD), USA [27]. BHRR is a multilevel intervention trial that offered corner stores discounts for healthier foods at a major wholesaler and promoted these foods through a targeted communications campaign at the corner store and wholesaler levels [27]. More than 63% of residents in the study areas are African American, and of these residents, 42.3% of adults are obese (BMI ≥ 30) [28,29]. Access to healthy food options is low in these neighborhoods, with a mean Healthy Food Availability Index (HFAI) score of 5.5 points (maximum 27 points) [30].

### 2.2. Study Sample

The study sample includes 24 corner stores from low-income areas of East and West Baltimore City. Store inclusion criteria were: (1) average purchases between $5000–$20,000 per year from wholesalers in 2009; (2) not part of past Baltimore Healthy Stores intervention trials [17,31]; (3) situated in low-income census tracts where greater than 75% of residents were African American, and (4) at least 0.25 miles apart from each other. Eighty-two stores were asked to participate in the study; 32 initially agreed to participate and out of those, eight dropped out of the study prior to baseline data collection, leaving 24 corner stores for the current analysis.

### 2.3. Defining Corner Stores’ Healthy versus Unhealthy Suppliers

We defined a healthy supplier as a store from which any of the 24 storeowners purchased at least one healthy food. Healthy foods were defined as BHRR’s promoted foods (Appendix, Table A1): Lower calorie beverages (e.g., bottled water, diet soda, low-fat milk); healthier essentials (e.g., whole wheat bread, canned tuna in water, frozen vegetables); and healthier snacks (e.g., fruits, baked chips), compared to items higher in salt, fat, and sugar (e.g., chips, candy, sugar sweetened beverages), which are more commonly stocked by corner storeowners [27].

Suppliers designated as unhealthy included those from which any of the 24 storeowners acquired unhealthy food options, such as chips, candy, baked goods, and sugar sweetened beverages. Classification as a healthy or unhealthy supplier were not mutually exclusive categories, given that storeowners may acquire healthy products at the same supplier from which they acquire unhealthy products. If a storeowner reported having shopped at a healthy supplier, the supplier was included in the HSN, regardless of whether or not the supplier also provided unhealthy products.

Each of the 24 corner storeowners provided information about all of their food and beverage suppliers by completing a 60-min interview. Interviewers asked storeowners to list the food and beverage suppliers they used: “In an average month, how many times do you (and/or your staff) shop for your store at the following?” and provided a list of the names of common suppliers as well as space to include names of suppliers not listed. Storeowners were also asked, “In the past 30 days, what food or beverage suppliers delivered directly to your store and how frequently?” and “Where did you buy (BHRR promoted food) in the last 30 days?” We then classified the type of supplier based on the U.S. Department of Labor, Standard Industrial Classification food store categories (*i.e.*, grocery store, wholesale, beverages, *etc.*) [32].

### 2.4. Defining Corner Stores’ Healthy versus Unhealthy Supplier Networks

The healthy supplier network (HSN) and unhealthy supplier network (UHSN) of corner stores was then defined based on corner storeowners’ sources of healthy and unhealthy foods and beverages (as described above). In other words, this information represented established networks and relationships between each of the 24 corner stores and their suppliers. The HSN consisted of suppliers from which the 24 corner storeowners reported acquiring healthy foods and beverages to stock in their store. Similarly, the UHSN consisted of suppliers from which the 24 corner stores reported acquiring unhealthy foods and beverages to stock in store. For instance, if a corner storeowner reported purchasing soda and chips from Wholesaler X and Supermarket Y, then Wholesaler X and Supermarket Y are part of that corner store’s UHSN. However, being classified as part of the UHSN does not exclude these suppliers from also being part of the HSN. Therefore, if the same corner storeowner purchased bananas and water from Wholesaler X but not from Supermarket Y, then Wholesaler X is part of that corner store’s HSN, but Supermarket Y is not. From these networks, we can characterize this corner storeowner’s supplier network and conclude that its UHSN includes Wholesaler X and Supermarket Y, while its HSN includes only Wholesaler X.

### 2.5. Supplier Network Analysis

Network analysis in the food context can help to formally characterize and compare the supplier networks of corner stores. In other fields, networks analysis has been used to elucidate and understand connections, such as those among people in healthcare organizations, and other institutions [33,34,35,36,37,38,39,40,41]. For example, one study examined the structure of multiple organizations implementing state tobacco control programs and found that networks with frequent communications resulted in greater productivity [41].

Our analysis utilized supplier connections to corner stores to create network diagrams (sociograms) representing the healthy and unhealthy supplier networks. For each supplier network, two sets of sociograms were generated: (1) two-mode network with connections between a supplier and a corner store. The connection is represented by an edge (line), each circular node represented one of the 24 corner stores, and each triangular node represented a supplier; and (2) one-mode network in which each edge (line) represents at least one common supplier shared between the two connected stores (circular nodes). It should be noted that the sociograms do not represent geospatial distances between corner stores and suppliers. Our analysis calculated the following measures for the HSN and UHSN of corner stores:

- Network core (two-mode sociogram): Set of suppliers that are central to each network, encompassing at least 80% of possible connections, or edges, between corner stores and suppliers [42]. Suppliers in the core indicate higher connectivity and importance in the network.
- Diversity (two-mode sociogram): Number of different types of suppliers connected to each corner store in the network; type of supplier was classified based on the U.S. Department of Labor, Standard Industrial Classification food store categories [32].
- Degree centrality (two-mode sociogram): Corner store’s number of existing connections with suppliers, expressed as a normalized percentage (number of existing connections divided by all possible connections with suppliers in the network). Higher degree centrality indicates greater connectedness between suppliers and corner stores in the network, and highly connected suppliers to corner stores may have greater influence, whereas relatively isolated suppliers with less connectedness may have little influence.
- Density (one-mode sociogram): Number of corner stores sharing at least one supplier, expressed as a percentage. Density can be interpreted as representing cohesion or the extent to which corner stores in a network are linked together through shared suppliers.

We used UCINET version 6.0 (Analytic Technologies, Lexington, KY, USA) to conduct the supplier network analysis.

### 2.6. Statistical Analyses

We assessed whether healthy and unhealthy supplier networks differed significantly in terms of degree centrality and density using T-tests. We also used bivariate regressions to assess whether store characteristics were associated with degree centrality of the unhealthy and healthy supplier networks. Since degree centrality represents corner stores’ established relationships with suppliers and part of network analysis is to understand factors that may be associated with storeowners establishing certain supplier relationships, we focused on degree centrality as the outcome variable; we relied on bivariate regressions due to the study’s small sample size (*n* = 24).

### 2.7. Bivariate Regression Analysis

#### 2.7.1. Dependent Variable

Degree centrality, which is a corner store’s number of existing connections with suppliers in a two-mode sociogram and expressed as a normalized percentage of all possible connections with suppliers in the network, was the outcome variable of interest. Degree centrality is important because it represents corner stores’ existing networks with suppliers, which is believed to have implications related to their access to unhealthy *versus* healthy suppliers, and consequently unhealthy *versus* healthy products, respectively.

#### 2.7.2. Independent Variables

Store-level characteristics of interest included: (1) corner stores’ average distance to each supplier; for suppliers with multiple distribution sites, we used the address of the distribution site closest to each corner store by using ArcGIS 10.1 (Environmental Systems Research Institute: Redlands, CA, USA, 2012); (2) Woman, Infants, and Children (WIC) acceptance; (3) Supplemental Nutrition Assistance Program (SNAP) acceptance; (4) storeowner ethnicity; (5) years of storeowner experience; (6) number of store customers reported on an average day; and (7) number of family and non-family employees. SNAP is the largest federal program in the U.S. that offers food assistance to low-income individuals and families. Households can use SNAP benefits to buy foods such as fruits and vegetables, meats, fish, dairy products, bread, and cereals [43]. WIC is a federal program that provides vouchers for foods to low-income pregnant, postpartum, and breastfeeding women and infants and children up to the age of 5. WIC benefits may cover foods such as fruits, vegetables, eggs, lower-fat milk, lower-fat cheese, and whole wheat bread [44]. Many corner stores in this setting accept SNAP and/or WIC benefits, making them an important source of nutritious food for low-income individuals and families. Asian or Hispanic storeowners were grouped together for this analysis due to the similarity in their HFAI scores, compared to African American storeowners, and due to the small sample size of Hispanic storeowners (*n* = 2) which was too few to be a stand-alone group.

Storeowners stock their stores through a combination of self-supply and delivery. Distance may become an issue when owners of corner stores, that are already limited in staff, must take the time to travel to various suppliers in order to stock their stores (*i.e.*, self-supply). Given that some of the suppliers in this study have multiple distribution locations, we assumed that storeowners purchased from the supplier located closest to the corner store. To account for distance and assess whether these travel distances affected connections in corner stores’ supplier networks, we performed a sensitivity analysis that eliminated suppliers which deliver to corner stores.

This study was approved by the Johns Hopkins Bloomberg School of Public Health Institutional Review Board. Statistical analyses were performed using Stata version 10 (StataCorp, College Station, TX, USA).

## 3. Results and Discussion

Table 1 provides a summary of corner store characteristics. The 24 corner storeowners listed a total of 42 different suppliers (Table 2), covering 7 supplier categories: wholesale club (*n* = 11); snacks (*n* = 10); other specialty foods, such as breads and coffee (*n* = 4); beverages (*n* = 5); supermarket/grocery (*n* = 9); discount department store (*n* = 2); and meat markets (*n* = 1).

### 3.1. Network Core Suppliers and Diversity of Suppliers

**Figure 1:**
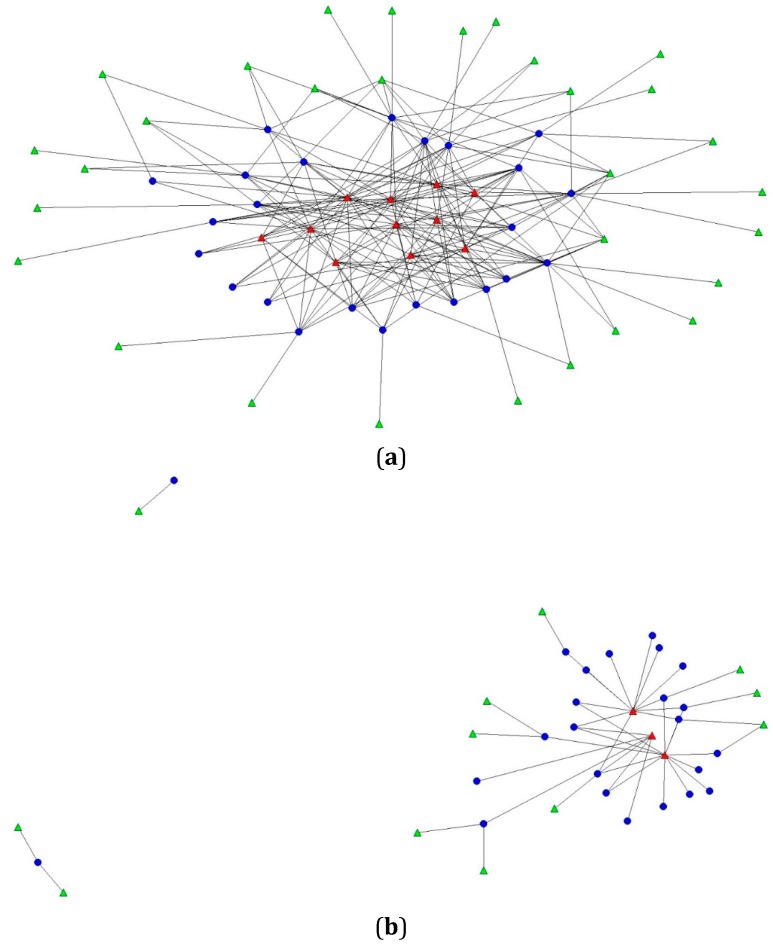
(**a**) Two-mode sociogram depicting relationships in corner stores’ unhealthy supplier network (UHSN) in Baltimore City, MD; triangles represent the food and beverage suppliers in corner stores’ UHSN (red triangles are core suppliers); circles represent the corner stores; edges (line) represent a connection between a corner store and a supplier; (**b**) Two-mode sociogram depicting relationships in corner stores’ healthy supplier network (HSN) in Baltimore City, MD; triangles represent the food and beverage suppliers in corner stores’ HSN (red triangles are core suppliers); circles represent corner stores; edges (line) represent a connection between a corner store and a supplier.

Figure 1a,b represent two-mode network sociograms representing corner store and supplier connectivity in the unhealthy and healthy supplier networks. In the UHSN (Figure 1a), we observed a network core of 11 suppliers. Core suppliers in the UHSN were diverse, with nine of the 11 core suppliers being a range of wholesalers (*n* = 5) and a variety of snacks suppliers (*n* = 4) that sell chips and baked goods; the remaining core suppliers being a discount store and beverage supplier. In contrast, the core of the HSN consisted of only three suppliers (Figure 1b). Similar to the UHSN, the HSN core comprised wholesalers (*n* = 2) and one discount store.

**Table 1:** Summary characteristics of sampled corner stores in Baltimore City, MD.

Corner Stores (*n* = 24)	Overall (mean ± SD)
Average distance to suppliers, km	34.0 ± 29.2
Accept WIC, yes	45.8%
Accept SNAP, yes	91.7%
Storeowner ethnicity	
African American	21.0%
Asian or Hispanic	79.0%
# years operating current store	9.5 ± 7.7
# years operating any food store	15.6 ± 9.1
# customers on an average day	165.0 ± 145.2
# non-family paid employees	0.75 ± 1.0
# family member employees	1.96 ± 1.3

**Table 2:** Names of suppliers in each supplier category. Reported by corner storeowners in Baltimore City, MD.

Unhealthy Supplier Network	Healthy Supplier Network
*Wholesale club*	
B. Green Wholesale East	B. Green Wholesale East
B. Green Wholesale West	B. Green Wholesale West
BJ’s Wholesale Club	BJ’s Wholesale Club
Costco Wholesale	Costco Wholesale
Eastern Food Services	Jetro Cash & Carry
George J. Falter Co.	Maryland Cash & Carry (2)
Jetro Cash & Carry	Sam’s Club
LG Wholesale	
Maryland Cash & Carry	
Restaurant Depot	
Sam’s Club	
*Snacks*	
Berliner Specialty Distributors (ice cream)	McKee Foods
Blue Bunny (ice cream)	Utz Quality Foods
Brigg’s Ice Cream Co.	
Frito Lay	
Herr’s Snacks	
McKee Foods	
Raylicious	
Stone Creek Countrywide Snacks	
Tastykake	
Utz Quality Foods	
*Other Specialty Foods*	
Farmer’s market	
Hauswald Bakery	
Sponseller’s Egg Co.	
Zeke’s Coffee	
*Beverages*	
Arizona Beverages USA	
Canada Dry	
Coca-Cola Company	
Everfresh Beverages	
PepsiCo	
*Supermarket/Grocery*	
Food Depot	Giant Food Stores
Giant Food Stores	Wegmans
Mars Supermarkets	Safeway
Martin’s Food Markets	Save-A-Lot
Safeway	
Save-A-Lot	
Shoppers Foods and Pharmacy	
Stop Shop and Save	
*Discount Department Store*	
Dollar Mark	Walmart
Walmart	
*Meat Market*	
Manger Packing Corporation	

*Notes*: The healthy supplier network (HSN) and unhealthy supplier network (UHSN) of corner stores was defined based on established networks and relationships between each of the 24 corner stores and their suppliers. The HSN consisted of suppliers from which the 24 corner storeowners reported acquiring healthy foods and beverages to stock in their store, such as lower calorie beverages (e.g., bottled water, diet soda, low-fat milk); healthier essentials (e.g., whole wheat bread, canned tuna in water, frozen vegetables); and healthier snacks (e.g., fruits, baked chips). The UHSN consisted of suppliers from which the 24 corner stores reported acquiring unhealthy foods and beverages to stock in store, such as chips, candy, baked goods, and sugar sweetened beverages. Classification as a healthy or unhealthy supplier was not mutually exclusive.

### 3.2. Degree Centrality

Overall, degree centrality was significantly greater in the UHSN (Figure 1a) than in the HSN (Figure 1b). In other words, corner stores’ number of existing connections with suppliers in the UHSN was nearly twice that of the HSN. Specifically, the normalized percentage of corner storeowners’ connections to suppliers in the UHSN was significantly greater (*t* = 5.23, *p* < 0.001) than in the HSN, 21.5% (95% CI 0.18 to 0.25) and 11.7% (95% CI 0.09 to 0.14), respectively.

### 3.3. Corner Store Characteristics Associated with Degree Centrality

For the UHSN, corner store characteristics were not significantly associated with degree centrality in our analysis (Table 3). However, for the HSN, SNAP acceptance and corner storeowner ethnicity were significantly associated with fewer ties to suppliers in the network. Corner stores that accepted SNAP benefits, compared to those that did not, had −1.864 (95% CI −3.067 to −0.660) fewer connections to healthy food suppliers (*p* = 0.004). Compared to African Americans, the Asian and Hispanic corner storeowners had on average −1.526 (CI −2.255 to −0.798) fewer connections to suppliers in the HSN (*p* < 0.001). Contrary to expectation, we found no relationship between WIC acceptance and degree centrality in the HSN.

**Table 3:** Bivariate analyses of corner stores’ unhealthy and healthy supplier networks, outcome variable being degree centrality.

Unhealthy Supplier Network (*n* = 41)			Healthy Supplier Network (*n* = 15)	
	b (95% CI)	*P*	b (95% CI)	*P*
Average distance to suppliers (km)	0.018 (−0.008, 0.044)	0.156	0.041 (−0.047, 0.128)	0.345
Accept WIC, yes	1.315 (−1.656, 4.286)	0.369	−0.287 (−1.086, 0.513)	0.465
Accept SNAP, yes	−0.182 (−5.639, 5.275)	0.946	−1.864 (−3.067, −0.660)	0.004
Storeowner ethnicity				
African American	ref	ref	ref	ref
Asian/Hispanic	0.295 (−3.417, 4.007)	0.871	−1.526 (−2.255, −0.798)	<0.001
# years operating current store	0.169 (−0.017, 0.355)	0.072	−0.006 (−0.060, 0.048)	0.818
# years operating any food store	0.104 (−0.059, 0.267)	0.198	−0.028 (−0.016, 0.071)	0.196
# customers on an average day	0.004 (−0.006, 0.015)	0.431	0.000 (−0.003, 0.002)	0.777
# non-family paid employees	0.311 (−1.241,1.863)	0.682	0.077 (−0.337, 0.493)	0.701
# family member employees	0.582 (−0.544, 1.708)	0.295	0.019 (−0.289, 0.328)	0.898

### 3.4. Sensitivity Analysis

Connections between corner stores and certain suppliers may be established as a result of convenience, given that some manufacturers often deliver products to corner stores. After accounting for suppliers who deliver to corner stores, therefore eliminating the need for storeowners to travel for certain products or self-supply, the relationship between independent variables and the outcome measure did not change (Appendix, Table A2).

### 3.5. Supplier Network Density

**Figure 2:**
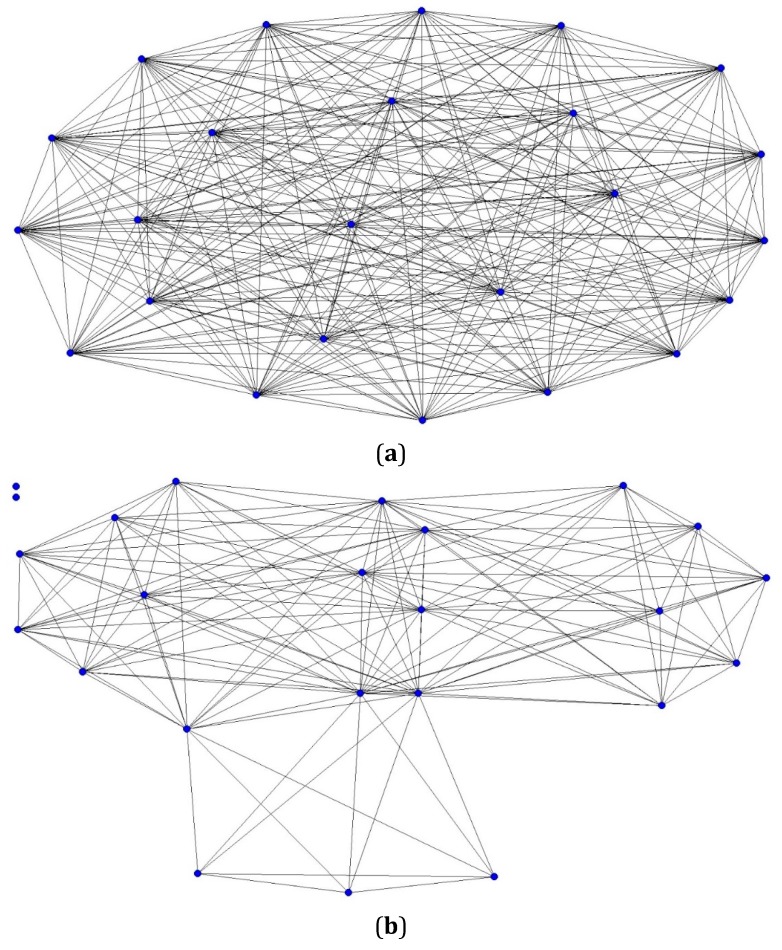
(**a**) One-mode sociogram depicting corner stores’ shared suppliers in the UHSN; Note: Circles represent corner stores; edges (line) represent at least one shared supplier between any given pair of corner stores; (**b**) One-mode sociogram depicting corner stores’ shared suppliers in the HSN. Note: Circles represent corner stores; edges (line) represent at least one shared supplier between any given pair of corner stores.

Supplier network density, which represents the extent to which corner stores are linked together through shared suppliers, of the UHSN was significantly different from the network density of the HSN (*t* = 5.82; *p* < 0.001) (Figure 2a,b). The network density of the UHSN was 99.6% (Figure 2a), and within this network, corner stores shared an average of nine suppliers (range: 3–15). In the HSN, we observed a much sparser network formation with a network density of 51.1% (Figure 2b), and within this network, corner stores shared an average of two suppliers (range: 1–4). While there were no isolated stores in the UHSN, we found two isolated stores that did not share suppliers with other stores in the HSN.

## 4. Discussion

To our knowledge, this is the first study to apply network analysis to gain a better understanding of the connections between urban corner stores and their suppliers, and to examine how differences in these relationships might have implications for storeowners’ access to healthy *versus* unhealthy food options to stock in store. While many efforts have been made to improve healthy food availability in underserved communities [23], challenges in sustaining these interventions, especially in corner stores, persist. One potentially critical knowledge gap relates to how interventions have yet to take into account corner stores’ connections with suppliers and characteristics of those established relationships.

Findings from this study highlight clear differences in the supplier network of corner stores that are critical to consider when encouraging storeowners to stock healthier food and beverage options. In particular, our analysis indicates that wholesale clubs are richly connected and central to both the healthy and unhealthy supplier networks in this setting. Additionally, the UHSN is far more diverse, including more supplier categories than the HSN. In terms of corner store characteristics associated with supplier networks, corner stores with Asian or Hispanic owners have fewer connections with healthy food and beverage suppliers, compared to corner stores with African American owners. Lastly, the UHSN for corner stores is much denser, indicating greater cohesion and capacity to spread resources wider and faster, than the HSN.

We speculate that corner stores’ healthy supplier networks may in part be limited because of storeowners’ misperception of consumer demand for healthier products. For instance, a mixed-methods study conducted with urban food storeowners and residents found that storeowners were reluctant to stock healthier food options due to perceived low consumer demand for healthier products, while residents reported feeling displeased with the limited range and quality of healthy food options in their local stores [45]. Moreover, other researchers have reported on the higher cost of food in corner stores, particularly to stock and sell healthy products, compared to larger retailers such as supermarkets [26,46,47]. As a consequence, healthy food options become less affordable for low-income residents in many food desert neighborhoods, which then further perpetuate the perception of consumer low demand for healthy products. As storeowners stock less healthy products, suppliers in turn perceive low demand for healthy options and experience little incentive to make healthy items more affordable and accessible to storeowners.

Our findings have several important implications. First, for readers unfamiliar with corner stores, it is important to understand that such food retailers are family-owned, small businesses operating with few staff members and small profit margins. Corner stores in this setting also have low levels of healthy food availability, with a mean HFAI score of 5.5 points (maximum 27 points) [30]. Our findings suggest that corner stores with fewer relationships with healthy food and beverage suppliers may have a smaller pool from which to shop, thereby making access to those options less convenient to stock and replenish [26]. Findings from other fields of study suggest that networks with more connections and greater density facilitated the spread of goods, services, support, and disease, suggesting that in the case of corner store supplier networks, increasing connectivity and density of the HSN might facilitate greater flow of these goods. For instance, individuals with larger social networks, which can increase social support or resources, experienced lower risk of family homelessness [48]. One way of addressing inequities in healthy food availability could be to improve the density or connectivity of the HSN to corner stores by establishing a “food hub” through which information about affordable healthy food options could be communicated and healthy food options could be distributed more efficiently [49].

Second, although corner stores are independent business entities, they have many core suppliers in common. Given storeowners’ concerns related to small profit margins, utilization of this shared connectedness between corner stores and suppliers suggests the possibility of group bulk-purchasing to lower the cost of food and beverages. Interestingly, in our network model, the wholesale club supplier category was seen as central to both the healthy and unhealthy food supply networks, making it a key player in the food supply chain to corner stores. Future studies can investigate ways to build on this existing connectivity to ensure an adequate supply of healthy options at the wholesale club level and promote the shift in sales from unhealthy to healthy products.

Third, in terms of supplier types, the UHSN is more diverse than the HSN, providing storeowners with more opportunities to acquire a range of less healthy food and beverage options. In particular, snack and beverage supplier categories were central in the UHSN but missing from the core of the HSN. Therefore, to improve sustainability of programs and policies requiring corner stores to stock healthier alternatives, support to storeowners should be provided to address the lack of connections with healthy suppliers. For example, information on the locations, delivery options, ordering methods, pricing, quality, and volume requirements for healthy food and beverage suppliers can help to guide planning and facilitate the process by which corner storeowners stock healthy products. Additionally, storeowners could also benefit from training and assistance with developing a business plan to ensure profitability and sustainability in stocking healthy products [50].

Fourth, storeowner ethnicity was significantly associated with the number of connections with healthy suppliers. Strategies to improve healthy supplier networks to corner stores may need to vary by storeowner ethnicity. Asian and Hispanic storeowners linked to fewer healthy suppliers may be a result of language and cultural barriers that hinder their ability to establish those relationships [51]. These findings suggest the need to consider storeowners’ capacity to navigate the food system when intervening at the retail level. Future work can further elucidate why and in what way certain relationships are established between corner stores and suppliers, and the level of connectivity that is needed for storeowners to cost-effectively and efficiently stock healthy food and beverages in stores.

Fifth, contrary to expectation, we found no relationship between corner stores’ acceptance of WIC benefits and degree centrality in the HSN. This outcome may be partly explained by the exclusion of some WIC-eligible foods (e.g., cheese, eggs, cereal) in this analysis, as BHRR’s intervention trial focused on beverages, snacks, and some essential foods such as whole wheat bread, fruits, and vegetables. Future research could include additional food and beverage products to further explore the supplier network of corner stores accepting WIC benefits. Finally, our results indicate that SNAP-accepting corner stores have fewer established healthy supplier connections compared to stores that do not accept SNAP. However, only two of the stores did not accept SNAP, so it is unclear what other differences may also be at work.

### Limitations

Our study has several limitations. First, BHRR recruited stores located in food deserts in low-income predominately African-American communities, which may limit the generalizability of the study’s findings. Furthermore, the response rate of storeowners was relatively low at 29.0%. Although the study sample might not be completely representative of corner stores in Baltimore City, approximately one in five city residents live in a food desert, and therefore working with and understanding the corner store food supplier network in these areas is of paramount importance. Second, there may have been other healthy food and beverage options outside of BHRR’s promoted foods, which if missed, could result in fewer suppliers in the HSN. However, healthy food availability in corner stores in this setting is very low, and BHRR’s list of promoted healthy options was extensive (see Appendix Table A1). The authors felt confident that storeowners’ report of suppliers from which these promoted foods were acquired would provide sufficient information to identify corner stores’ healthy suppliers. Third, measurement of distances between corner stores and suppliers assumed that storeowners accessed suppliers that were nearest to their stores. We also assumed that a given supplier with multiple distribution locations offered similar healthy and unhealthy products. Therefore, future studies would benefit from further analyzing storeowners’ acquisition of healthy *versus* unhealthy products, accounting for variability in product availability across multiple locations of the same supplier. In addition, storeowners in Baltimore City may purchase products at various suppliers located in route between their place of residence and work. However, because we did not ascertain storeowners’ home addresses, their store addresses were used for the purposes of this analysis. If storeowners also acquire products at suppliers near their place of residence, future analyses should also consider distances between suppliers and storeowners’ home addresses. Fourth, our supplier network analysis did not include the frequency of sourcing food and beverages from suppliers or the dollars spent at each supplier; such information could further characterize corner stores’ healthy and unhealthy supplier networks. Lastly, the limited sample size did not permit analyses with multivariate regression models, which could have helped to control for potential confounding and assess interactions. Future studies should include a larger sample size to allow for more complex analyses.

## 5. Conclusions

Disparities exist between the unhealthy and healthy supplier networks of corner stores in urban, low-income neighborhoods of Baltimore City, MD, USA. Applying social network principles to the food supply chain may assist researchers and policymakers in designing systems interventions at the supplier level to improve the food environment. To expand the availability of healthy options, efforts are needed to increase the diversity, degree centrality, and density of corner stores’ healthy supplier networks, while also considering needs that may vary by storeowner native language and ethnicity. Additionally, in urban low-income neighborhoods that are saturated with unhealthy food options, food policies that mandate healthy food access may not be sufficient to create positive changes in the food environment. Interventionists and policymakers must also take into consideration the existing supplier network and the language and cultural barriers that may hinder a storeowner’s ability to establish necessary relationships.

## Appendix

**Table A1:** List of BHRR promoted foods (from BHRR’s Store Impact Questionnaire).

Food	Standard Unit
Phase 1: Healthy Beverages	
1% Milk, Rutters	Half Gallon
1% Milk, Rutters	Gallon
1% Milk, Rutters	Quart
Bottled Water, Deer Park	16.9 fl oz bottle
Bottled Water, Deer Park	23.7 fl oz bottle
Pepsi Next	1 bottle (24 fl oz)
Coke Zero	1 bottle (24 fl oz)
Phase 2: Healthy Essentials	
Solid White Albacore Tuna in water, Bumblebee	1 can (5 oz)
Solid White Albacore Tuna in water, Starkist	1 can (5 oz)
Chunk Light Tuna in water, Bumblebee	1 can (5 oz)
Chunk Light Tuna in water, Starkist	1 can (5 oz)
100% Whole Wheat Bread, “Schmidt Old Tyme”	1 Loaf
Frozen Stir-Fry Vegetables, Bird’s Eye	1 bag (12 oz)
Frozen Collards, Bird’s Eye	1 bag (12 oz)
Frozen Broccoli, Bird’s Eye	1 bag (12 oz)
Frozen Vegetables Type:	
Frozen Vegetables Type:	
Frozen Vegetables Type:	
Frozen Vegetables Type:	
Frozen Vegetables Type:	
Frozen Vegetables Type:	
Phase 3: Healthy Snacks	
Apples, Gala	1 fruit
Bananas	1 fruit
Oranges, Navel	1 fruit
Fresh Fruit Type:	1 fruit
Fresh Fruit Type:	1 fruit
Fresh Fruit Type:	1 fruit
Fresh Fruit Type:	1 fruit
Baked Potato Chips, Assorted Utz	1 oz bag
Baked Potato Chips List:	
Baked Potato Chips List:	
Granola Bar, Quaker Oats 90 calorie, Assorted	1 bar
Granola Bar, Other Type:	1 bar

**Table A2:** Bivariate analyses of corner stores’ supplier networks, outcome variable being degree centrality; excludes delivering suppliers.

**Unhealthy Supplier Network (*n* = 41)**				
	**All Suppliers**		**All Suppliers—Deliverers**	
	**b (95% CI)**	***P***	**b (95% CI)**	***P***
Mean distance (km)	0.018 (−0.008, 0.044)	0.156	0.134 (−0.206, 0.474)	0.422
Accept WIC, yes	1.315 (−1.656, 4.286)	0.369	0.622 (−0.628, 1.873)	0.313
Accept SNAP, yes	−0.182 (−5.639, 5.275)	0.946	−0.864 (−3.140, 1.413)	0.440
Storeowner ethnicity				
African American	ref	ref	ref	ref
Asian/Hispanic	0.295 (−3.417, 4.007)	0.871	0.011 (−1.560, 1.581)	0.989
Time operating current store (years)	0.169 (−0.017, 0.355)	0.072	0.039 (−0.044, 0.122)	0.339
Time operating food stores (years)	0.104 (−0.059, 0.267)	0.198	0.002 (−0.069, 0.074)	0.945
No. of customers on average day	0.004 (−0.006, 0.015)	0.431	0.003 (−0.001, 0.007)	0.148
No. of non-family paid employees	0.311 (−1.241,1.863)	0.682	0.189 (−0.465, 0.842)	0.555
**Healthy Supplier Network (*n* = 15)**				
	**All Suppliers**		**All Suppliers—Deliverers**	
	**b (95% CI)**	***P***	**b (95% CI)**	***P***
No. of family member employees	0.582 (−0.544, 1.708)	0.295	0.078 (−0.409, 0.565)	0.742
Mean distance (km)	0.041 (−0.047, 0.128)	0.345	−0.048 (−0.161, 0.065)	0.391
Accept WIC, yes	−0.287 (−1.086, 0.513)	0.465	−0.224 (−0.926, 0.479)	0.516
Accept SNAP, yes	−1.864 (−3.067, −0.660)	0.004	−1.455 (−2.560, −0.349)	0.012
Storeowner ethnicity				
African American	ref	ref	ref	ref
Asian/Hispanic	−1.526 (−2.255, −0.798)	<0.001	−1.432 (−2.029, −0.834)	<0.001
Time operating current store (years)	−0.006 (−0.060, 0.048)	0.818	−0.023 (−0.069, 0.022)	0.302
Time operating food stores (years)	−0.028 (−0.016, 0.071)	0.196	0.015 (−0.024, 0.054)	0.426
No. of customers on average day	0.000 (−0.003, 0.002)	0.777	0.000 (−0.003, 0.002)	0.645
No. of non-family paid employees	0.077 (−0.337, 0.493)	0.701	0.044 (−0.320, 0.409)	0.803
No. of family member employees	0.019 (−0.289, 0.328)	0.898	0.041 (−0.229, 0.311)	0.758
